# Bayesian smoothed small-areas analysis of urban inequalities in fertility across 1999–2013

**DOI:** 10.1186/s40738-019-0066-8

**Published:** 2019-12-21

**Authors:** Brenda Biaani León-Gómez, Mercè Gotsens, Marc Marí-Dell’Olmo, Ma. Felicitas Domínguez-Berjón, Miguel Ángel Luque-Fernandez, Unai Martin, Maica Rodríguez-Sanz, Gloria Pérez

**Affiliations:** 1 Sistemes d’Informació Sanitària, Agència de Salut Pública de Barcelona, Barcelona, Spain; 2 Department of Experimental and Health Sciences, Universitat Pompeu Fabra, Barcelona, Spain; 3 Institut de Recerca Hospital de la Santa Creu i Sant Pau, Barcelona, Spain; 4 Centro de Investigación Biomédica en Red de Epidemiología y Salud Pública (CIBERESP), Madrid, Spain; 5 Qualitat i Intervenció Ambiental, Agència de Salut Pública de Barcelona, Barcelona, Spain; 6 Dirección General de Salud Pública, Consejería de Sanidad, Madrid, Spain; 7 Faculty of Epidemiology and Population Health, Department of Non-Communicable Disease Epidemiology, London School of Hygiene and Tropical Medicine, London, UK; 8 Departamento de Sociología 2, Universidad del País Vasco UPV/EHU, Leoia, Bizkaia Spain; 9 Recerca, Docència i Comunicació, Agència de Salut Pública de Barcelona, Barcelona, Spain

**Keywords:** Fertility, Small-areas, Social inequalities, Urban health, Economic recession, Immigration

## Abstract

**Background:**

Since the 2008 economic crisis in Spain, overall fertility has continued to decrease, while urban inequalities have increased. There is a general lack of studies of fertility patterns in small-areas of Spanish cities. We explored the effects of the economic crisis on fertility during three time periods in urban settings in Spain.

**Methods:**

We studied the distribution of fertility rates among women (15–49 years) from Spain and low-middle income countries (LIC) who were living in 13 Spanish cities. We mapped fertility and the MEDEA socioeconomic deprivation index in small-areas, and analyzed age-related trends in fertility rates. We performed an ecological regression analysis of fertility and the deprivation index in two pre-crisis periods (1999–2003 and 2004–2008) and one crisis period (2009–2013). Fertility rates were calculated and smoothed using the hierarchical Bayesian model (BYM).

**Results:**

Higher fertility was generally associated with socioeconomic deprivation, with adjustment for the mothers’ age and nationality. While Spanish citizens tended to delay childbearing throughout the three study periods, fertility increased among Spanish adolescents from deprived urban areas during the economic crisis. There was a general decline in fertility among immigrants after the crisis, especially in southern cities. Overall, fertility appeared to be stable, with higher fertility in more deprived areas.

**Conclusion:**

Increased unemployment and changes to government family policies may have contributed to delayed childbearing in Spain. For immigrants, more restrictive immigration policies may have played a crucial role in decreasing fertility rates. Reforming such policies will be key for better reproductive rights and improved fertility rates across all population cohorts in Spain.

## Background

Fertility refers to the rate of production of offspring and is dependent on several diverse factors: the socioeconomic and political context of the country (government welfare policies); social (ethnicity, age, social class); labor-related (employment status and working conditions); and psychosocial and biological factors, such as infertility or individual choices [1, 2]. Health inequalities tend to be more marked in urban areas where deprived and poor populations live [3, 4]. Small-area studies can bridge the understanding between social policies and their health implications, as particular clusters and patterns may not be as discernible in larger area studies [5–7]. Within this type of study, area effects refer to physical and social factors that may affect public health. Examples include urban planning and the provision of public and private services, which may be important contributors to health inequalities. Further, demographic trends in small-areas are shaped by several key factors, such as declining fertility rates, an ageing population, migration, and the socioeconomic landscape [8].

Fertility rates in Spain have been decreasing since the 1970s, stabilizing around 2000, and continuing to decrease since 2008 [9–11]. Previous studies have described the influence of the economic crisis on fertility in Spain [11, 12], where the strongest effects of the crisis were felt among the most vulnerable population groups [13, 14]. Women and the young population were particularly hard hit by high unemployment rates [15]. The crisis altered the socioeconomic landscape, resulting in various political responses and policy changes [16]. Some of these changes weakened the Spanish welfare system, thereby affecting many structural determinants of health. Among others, gender equality policies and family policies were subject to drastic cuts, institutions and government bodies created to promote gender equality have been dismantled or downgraded, and some policies such as the paternity benefit has not yet been implemented after it was frozen during the crisis. In this sense, Spanish family policies were negatively affected, thereby increasing barriers for people to raise children [17].

As far as we are aware, the clusters and patterns of fertility have not been analyzed at the level of small-areas in this country. Following the social changes brought about by the crisis in Spain, there is a need to study fertility at the territorial level, and the role played by associated inequality axes such as income and migration. Therefore, the aim of this study is to explore the effect of the economic crisis on the distribution of fertility across small-areas in urban settings in Spain and to consider the role of mothers’ age and nationality.

## Methods

### Design, unit of analysis and study population

As part of the *IMCRISES* project, we conducted an ecological study of trends during three periods: 1999–2003, 2004–2008 (pre-crisis periods) and 2009–2013 (crisis period), where 2009 was considered as the year when the economic crisis started in Spain [15, 18]. The units of analysis were the census tracts of 13 Spanish cities, as defined in the 2001 Spanish Population and Housing Census.

The cities included in the study are located in different geographical regions of Spain: Madrid (Additional file 2 and 3); Barcelona (the second most populous city, located in northeast); eight cities in the most southern region, Andalusia (Seville, Almeria, Cadiz, Cordoba, Granada, Huelva, Jaen, and Malaga), and three cities from a north-west region, the Basque Country (Bilbao, San Sebastián, and Vitoria). The study population comprised women of reproductive age who were living in these cities between 1999 and 2013.

### Information sources

We obtained birth data from the official birth records of the respective cities. Postal addresses, ages and nationalities of the mothers were sourced from the National Institute of Statistics. We geo-coded the postal addresses to obtain the census tract. Data on the number of reproductive-aged women, which was defined as 15 to 49 years [19], were obtained from the city register of inhabitants. We used the socioeconomic index that was formulated and used by the *MEDEA* project [20]. The deprivation index for each city was defined as that from 2001 Population and Housing Census.

### Description of the variables

The fertility rate was calculated as the number of live births per 1000 women of childbearing age (15 to 49 years) during each study period. We stratified all analyses by period, age, and nationality of the mother. Five age groups were analyzed: 15–19; 20–24; 25–34: 35–49; 15–49 years. In this study, Spanish nationals and people from high-income countries were grouped into a single category (Spanish women) for analysis. Individuals from low-and-middle-income countries were grouped into one category [women from low-income countries (LIC)]. High-income countries were those with a gross national per capita income of ≥$12,056 (see list of high income countries in the World Bank website) [21].

Based on previously described methods [20], we included the MEDEA deprivation index as a covariate, the principal components of this index were five socioeconomic indicators for each census tract: (a) manual workers: percentage of employed people aged ≥16 who are manual workers; (b) unemployment: number of people aged ≥16 years we are unemployed or actively seeking a job as a percentage of the total economically active population; (c) temporary workers: percentage of employed people aged ≥16 years where were employed in temporary jobs; (d) low educational level: percentage of people aged ≥16 years who have < 5 years of schooling or who did not complete basic compulsory education; (e) Low educational level in young people (16–29 years) [20]. Higher index values corresponded to greater deprivation, and vice versa. The index was normalized to a mean of 0 and standard deviation of 1. The index accounted for over 75% of the variability of the indicators in all cities.

### Statistical analysis

The dependent variable was fertility rate, as fertility depends on population size. However, fertility variance is inversely proportion to the expected values. Thus, areas with low population tend to have larger estimated variability. We used the hierarchical Bayesian model proposed by Besag, York and Mollié (BYM) to smooth the rate [22]. The model considers two types of random effects: spatial and heterogeneous random effects. The former concerns the spatial structure of the data, while the latter deals with non-structural (non-spatial) variability. We estimated the fertility rate for each period, age group and nationality. We used maps to represent the geographical distribution of the smoothed rates and deprivation. All maps were generated using the R statistical package [23]. We considered deprived areas to be those with the highest deprivation index (lowest septile of deprivation) of the MEDEA index in each city.

We used a regression model to analyze the association between fertility and deprivation during the three periods. Deprivation, interactions between periods and their random effects were also taken into account (see the model in the methodological annex). Regression models provided us with relative risks and their respective intervals. Changes in the deprivation and fertility associations were evaluated through the included interactions. Specifically, we have studied changes between the first and second periods, and the second and third periods. Changes between periods in the relationship between the socioeconomic deprivation index and mortality were evaluated through the interactions between the periods. Specifically, we studied the change between the first and second pre-crisis periods, and between the second pre-crisis period and the crisis period. All analyses used the Integrated Nested Laplace Approximations (INLA) method (INLA package) from the R (R.3.1.1) [24]. Details of the method can be found in the Additional file 1.

## Results

### Fertility rates

Table 1 shows crude fertility rates among Spanish and LIC women for each age group, time period, and city. In Spanish women, there appears to be a pattern of declining crude fertility rates in large cities and in southern Spain from the second to the third crisis period, except in the 35–49 years age group. In Spanish women, global (15–49) fertility rates in Vitoria and San Sebastián did not decline between the same periods. While LIC women had the highest fertility rates, global (15–49) decreases were observed in Madrid and in all cities in the South and Bilbao from the second pre-crisis period to the crisis period. Meanwhile, from the second pre-crisis period to the crisis period rates seemed to increase in Barcelona, Vitoria, and San Sebastian.

**Table 1:** Fertility rates in women from^a^ Spain and low-income countries (LIC) for each study period, region and city

Region	City	Age	Pre-crisis 1^st^ 1999–2003		Pre-crisis 2^nd^ 2004–2008		Crisis 2009–2013	
			Spanish	LIC	Spanish	LIC	Spanish	LIC
			Age specific fertility Rate		Age specific fertility Rate		Age specific fertility Rate	
North	Bilbao	15–19	5.11	31.45	6.62	39.66	5.91	30.01
		20–24	11.73	69.47	13.05	83.38	11.81	79.35
		25–34	61.00	50.50	64.17	72.73	59.50	68.39
		35–49	21.14	14.38	25.05	18.64	29.17	23.00
		15–49	30.59	38.06	33.68	50.74	33.59	48.07
	San Sebastian	15–19	1.90	24.62	2.32	32.78	1.58	26.07
		20–24	6.50	44.15	6.02	57.47	6.31	49.12
		25–34	75.28	42.55	72.81	56.08	71.07	58.89
		35–49	23.05	12.38	26.86	16.52	30.22	22.25
		15–49	34.68	28.38	35.57	39.09	35.94	40.87
	Vitoria	15–19	3.50	32.43	3.58	43.43	4.17	32.39
		20–24	10.37	77.89	10.13	102.98	10.28	86.71
		25–34	69.60	60.48	69.26	86.06	71.18	105.09
		35–49	19.28	18.01	24.54	24.37	30.05	34.59
		15–49	32.16	44.08	35.24	61.86	38.17	69.12
Largest Cities	Barcelona	15–19	3.22	28.83	4.56	21.89	3.60	17.30
		20–24	10.03	58.63	13.58	41.76	10.23	44.70
		25–34	64.28	60.52	65.63	48.03	59.78	49.94
		35–49	22.16	19.31	25.70	20.71	30.55	22.78
		15–49	31.70	41.16	34.84	35.58	34.29	36.25
	Madrid	15–19	5.78	58.81	6.082	35.78	4.81	19.40
		20–24	12.57	97.17	12.45	62.54	10.16	36.20
		25–34	70.40	70.92	62.58	52.36	52.85	40.78
		35–49	24.98	17.29	25.43	15.30	26.87	14.42
		15–49	36.55	54.95	40.03	54.58	39.83	47.53
South	Almeria	15–19	15.24	65.38	17.28	57.57	14.68	33.21
		20–24	35.89	180.32	38.11	123.60	30.95	112.22
		25–34	77.09	181.96	79.46	104.07	77.33	94.37
		35–49	18.27	41.82	21.44	30.46	24.21	29.16
		15–49	38.23	119.01	40.62	78.44	39.26	66.86
	Cádiz	15–19	8.22	14.29	10.27	18.63	6.36	18.32
		20–24	18.13	93.33	22.10	52.47	19.31	61.36
		25–34	56.20	54.12	59.85	66.67	56.45	63.98
		35–49	16.44	16.95	19.74	21.44	21.15	20.59
		15–49	27.38	45.40	31.20	44.23	29.77	43.62
	Córdoba	15–19	9.38	40.82	10.94	49.06	9.27	30.17
		20–24	21.14	100.12	26.88	86.62	25.69	78.40
		25–34	83.22	118.58	84.53	85.43	77.17	61.97
		35–49	19.01	38.27	21.82	27.74	24.66	19.10
		15–49	37.71	80.17	40.40	62.21	38.23	45.41
	Granada	15–19	11.20	33.96	11.79	40.78	8.53	24.48
		20–24	21.16	65.08	23.89	98.66	19.80	80.00
		25–34	67.11	89.19	65.18	98.33	59.51	95.19
		35–49	19.92	35.57	22.79	32.36	26.42	34.02
		15–49	32.88	64.32	34.20	71.84	33.15	65.82
	Huelva	15–19	11.87	35.21	15.86	41.98	12.35	27.80
		20–24	26.84	92.52	34.14	96.25	30.63	70.25
		25–34	80.52	90.07	80.54	91.27	77.72	69.38
		35–49	16.62	26.43	21.14	28.94	24.68	22.22
		15–49	37.41	67.37	41.40	67.35	40.19	47.75
	Jaen	15–19	7.09	56.91	11.50	55.94	10.12	14.93
		20–24	21.90	150.41	23.88	97.27	22.98	84.41
		25–34	91.07	144.40	87.72	87.30	78.81	76.22
		35–49	20.50	44.60	22.45	35.63	25.04	28.64
		15–49	40.33	99.13	40.72	69.09	37.90	55.10
	Malaga	15–19	12.89	33.13	15.19	38.71	11.83	23.71
		20–24	27.33	107.18	32.57	96.25	30.86	60.95
		25–34	79.13	143.04	80.17	100.03	74.87	68.24
		35–49	17.84	36.59	21.94	27.94	25.05	23.79
		15–49	37.59	85.54	40.83	68.25	38.99	47.45
	Sevilla	15–19	9.94	28.78	12.30	34.71	9.02	23.54
		20–24	20.18	95.13	25.79	79.86	25.65	66.32
		25–34	75.59	109.62	75.96	73.20	71.78	64.17
		35–49	20.45	29.80	25.19	23.36	27.94	21.73
		15–49	36.71	70.01	40.24	53.22	38.69	45.44

Age specific fertility Rate: the number of live births per 1000 women of childbearing age (ages 15 to 49) occurring in each study period. For the calculus, we used all the childbirths of each of the 4 years included in the study period (1999–2003; 2004–2008; 2009–2013) and the same for the population

*LIC* Women with a nationality from low-income countries

^a^According to nationality

### Distribution of fertility rates and deprivation index across small-areas in the 35–49 years age group

**Fig. 1:**
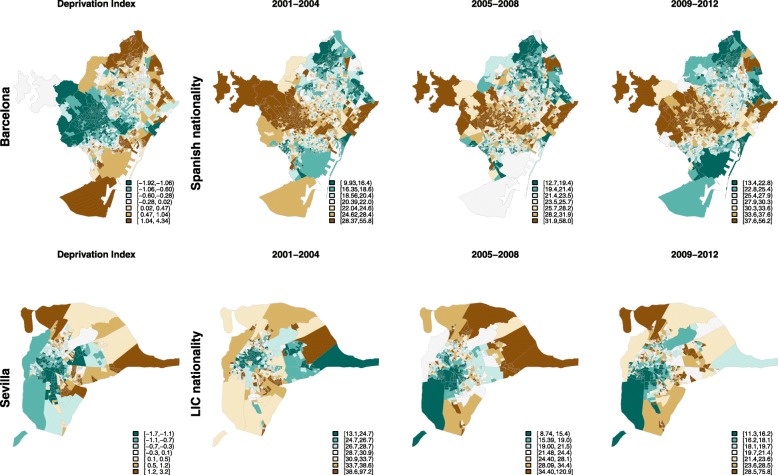
Smoothed fertility rates in small-areas (35- to 49-year-olds) among women from Spain living in Barcelona and women from low income countries (LIC) living in Seville, 1999–2013. * According to nationality. LIC: Women with a nationality from low-income countries

Figure 1 shows the distribution of the deprivation index in small-areas, as well as fertility rates in 35- to 49-year-old Spanish women (e.g. Barcelona) and women from LIC (e.g. Seville). Among Spanish women, there was a general pattern of lower fertility in deprived areas and higher fertility in affluent areas, for example in Barcelona. In contrast, fertility rates among women from LIC showed the inverse pattern: higher fertility in deprived areas and lower fertility in affluent areas, such as in Seville. These patterns did not change markedly during the crisis period. Stratified maps of other cities are shown in the Additional file 4.

### Differences in fertility between small-areas among Spanish women

**Fig. 2:**
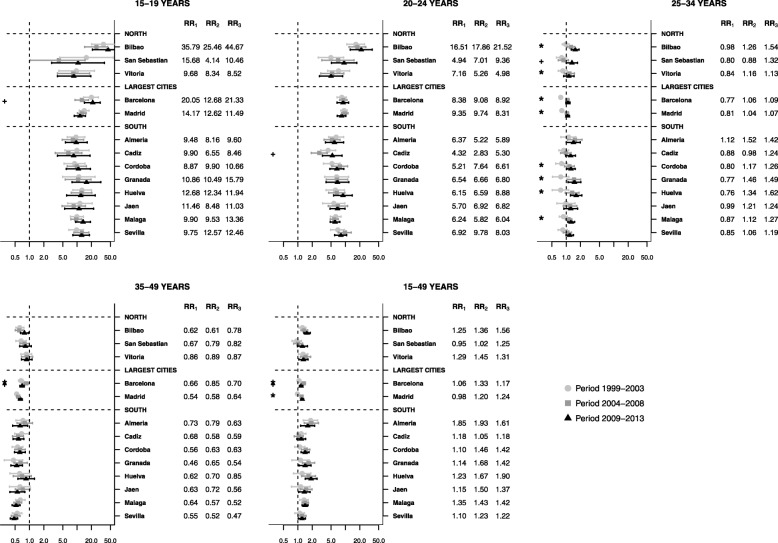
Association between fertility rate and the MEDEA deprivation index in Spanish women. Relative risk comparing 95th to 5th percentile of deprivation index for each age group, city and period, 1999–2013. RR_1_ = Relative risk of the first pre-crisis period (1999–2003). RR_2_ = Relative risk of the first pre-crisis period (2004–2008). RR_3_ = Relative risk of the first pre-crisis period (2009–2013). * Statistically significant difference from RR_1_ to RR_2._ + Statistically significant difference from RR_2_ to RR_3_

Figure 2 shows the association between fertility rates and the deprivation index among Spanish women, stratified by age, city and time period. There was a general positive association between fertility rate and the deprivation index among younger individuals, i.e. higher fertility among more deprived groups. This association appeared stronger among adolescents during the crisis. This was especially apparent in Barcelona (relative risk for the second pre-crisis period (RR_2_) 12.68; relative risk for the crisis period (RR_3_) 21.33), and could indicate a rise in inequality in certain areas.

In global across cities, the crisis did not appear to modify the association among 20- to 24-year-olds, whereas among 25-to-34-year-olds. Therefore, the differences in fertility rates between deprived and affluent areas increased among 25–34 year olds from the first to the second pre-crisis period. This positive association decreased or remained stable during the crisis. In contrast to the 15–19 years group, there was a negative association among 35–49-year-olds, i.e. fertility was higher in privileged areas and remained so throughout the crisis. The negative association remained stable during the crisis, except in Barcelona (RR_2_ 0.85 to RR_3_ 0.70) where it decreased further.

There was no change in fertility inequalities across the three periods in the southern and northern regions. In Barcelona, however, the associations between fertility and deprivation became weaker during the crisis (from RR_2_ 1.33 to RR_3_ 1.17). In contrast, there was positive association in Madrid from the first to the pre-crisis periods (RR_1_ 0.98 to RR_2_1.20).

### Differences in fertility between small-areas among women from LIC

**Fig. 3:**
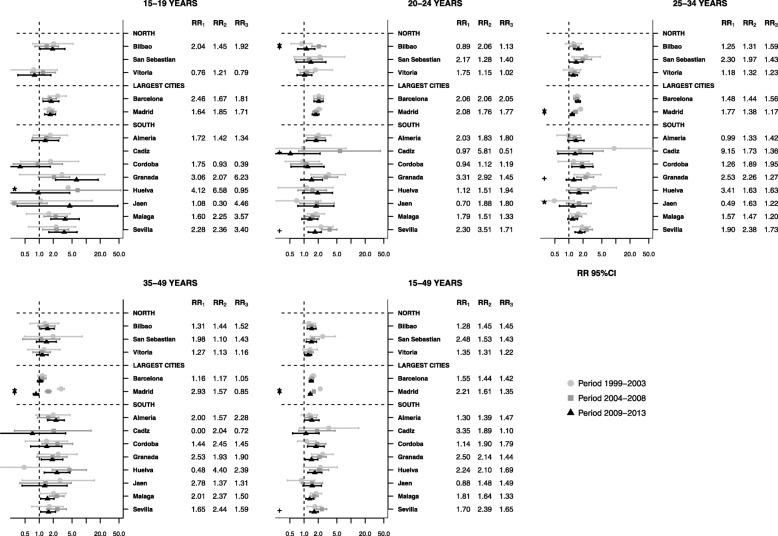
Association between fertility rate and the MEDEA deprivation index in women from low income countries (LIC). Relative risk comparing 95th to 5th percentile of deprivation index for each age group, city and period, 1999–2013. LIC: Women with a nationality from low-income countries. RR_1_ = Relative risk of the first pre-crisis period (1999–2003). RR_2_ = Relative risk of the first pre-crisis period (2004–2008). RR_3_ = Relative risk of the first pre-crisis period (2009–2013). *Statistically significant difference from RR_1_ to RR_2._ + Statistically significant difference from RR_2_ to RR_3_

There was a positive association between fertility and deprivation for all LIC women (Fig. 3), among whom fertility increased in deprived areas across all age groups and most cities studied. However, this seemed to decrease during the crisis in almost all cities. Only Madrid and Seville showed significant changes from RR_1_ to RR_2_. LIC women aged 35–49 years showed a positive association between deprivation and fertility. However, Madrid was an exception where the association diminished from the first pre-crisis period to the crisis period (RR_2_ 2.93 to RR_3_ 0.85).

The positive association between fertility rate and deprivation index among LIC women was generally consistent in different age groups and in most cities studied. Interestingly, the rate did not change markedly during the crisis. However, the association decreased in LIC women aged 20–24 years in Seville, Cadiz and Bilbao, 25–34 years in Granada, and 25–34 and 35–49 years in Madrid.

## Discussion

This study explores socio-economic inequalities in the distribution of fertility in 13 cities in Spain, where fertility rate was general associated with deprivation. However, the distribution varied according to the age and nationality of mothers. Among Spanish adolescents in disadvantaged areas, fertility appeared to increase during the crisis period, and this was also the case for Spanish women older than 34 years from more affluent areas. LIC women were found to have a stable positive association across the three periods, in which the pattern of higher fertility in more deprived areas remained unchanged.

Previous studies have noted that fertility in Spain may have responded negatively to the economic downturn [12, 25]. We observed a change in the distribution of fertility among 25- to 34-years-old Spanish women, the group with the highest fertility. Prior to 2003, the 25–34 years group had the highest fertility rate in affluent areas, after which a decline in fertility in this group during the crisis period. After 2004, the trend shifted and fertility increased in deprived areas. This is consistent with research showing that fertility rates decline with increased unemployment [26–31]. As in other European countries with higher fertility rates, this reversal may be linked to reduced availability of assistance for mothers due to difficult socio-economic circumstances [9].

For instance, the distribution of fertility among Spanish adolescents in Barcelona became more unequal during the crisis. We observed variation in the distribution of fertility in small-areas, where Spanish adolescents from more affluent areas had lower fertility rates, while rates increased in more deprived areas. This may be associated with an increase in unintended pregnancies among adolescents in disadvantaged areas. This trend seemed to be present in other cities in this study. The variation also suggests that the decrease in fertility in this group could be greater in affluent areas. In the case of Barcelona, other proxy indicators suggest the increase in the differences, such as decrease in the use of condoms by adolescents from disadvantaged social classes [32]. Pregnancy in adolescents in deprived areas is a concern, as it is one of the main contributors to the circle of disease and poverty [33]. Targeted health policies and campaigns could help to reduce adolescent pregnancies.

We found that Spanish women older than 35 years were less likely to have children if they lived in deprived areas, in spite of the economic crisis. Simultaneously, we observed a general trend of delayed childbearing in privileged areas among Spanish women, which is concordant with the overall increase in the average year at conception in Spain [9]. This delay may also be mediated by other factors such as unemployment and poor reconciliation between work and maternity leave, which may have been exacerbated by weakened family policies [9, 11, 34]. Last, empowerment of women and social changes could be an important influencing factor of delayed childbearing [35].

Immigrant women living in deprived areas were more likely to have children, which may be due to the higher density of immigrant populations in deprived areas. In the south (Andalusia), the region most affected by the recession and unemployment, fertility among immigrants decreased [36]. Economic decline and unfavorable working conditions may have been associated with decreased fertility among LIC women. Working conditions in this population may be more difficult due to legal obstacles (such as difficulties in obtaining a work permit). This in turn may be linked to employment instability and diminished health rights. Following reforms of immigration laws in Spain, rights to free healthcare for irregular immigrants were stopped [37, 38]. Therefore, it is possible that such policies may affect the reproductive rights of this population. These factors may create uncertainty, which may lead some women to make different pregnancy decisions.

The most susceptible population groups are immigrants without full Spanish citizenship rights. Therefore, in this study it was more meaningful to address nationality rather than country of origin. However, data on nationality were not available for all participants, the limitation being that people from LIC could also hold another nationality [39]. We analyzed the available data on double nationality in mothers (these data were only available for Barcelona) and found that 21% of mothers from LIC reported that they also had Spanish nationality, representing 9% of all mothers with Spanish nationality. Ultimately, this is an important strength because it brings us closer to the most vulnerable population (those without all citizens’ rights), since women who have already obtained Spanish nationality have probably lived in Spain for longer, with the additional rights that this brings. Another limitation was that we combined women who were not from low- and middle-income countries in one group, thereby combining several and different countries. Last, we determined unequal distribution using area and socioeconomic differences. While this carries important limitations, it was not possible to encompass all determinants of fertility in this current study.

## Conclusion

This study observed a general decline in fertility among women in resource-deprived regions in Spain, which may be associated with the economic recession. Southern Spain, the region with the highest rates of unemployment, showed the greatest decrease in fertility after the crisis, especially among immigrant women from LIC. Restrictive immigration policies may have affected the fertility of LIC women. Inequality tended to increase over time and, similarly, the economic crisis appeared to affect socioeconomic inequalities in fertility among Spanish adolescents. Adolescents living in deprived areas had higher fertility rates, due in part perhaps to an increase unintended pregnancies. In contrast, adult Spanish women from all regions tended to delay childbearing. More accommodating pro-family policies and increased employment will likely help generate improved working and living conditions, to give women in Spain more freedom in deciding when to have a child.

## Supplementary information

**Additional file 1.** Supplementary methods

**Additional file 2.** Supplementary contextual factors of the cities

**Additional file 3.** Supplementary file cities map

**Additional file 4.** Supplementary results

## Data Availability

The data is available through request via email to the corresponding author.

## Abbreviations

BYM: Besag York Mollie
IMCRISES: The effect of the economic crisis on sexual and reproductive health, and socioeconomic inequalities in Spain
INLA: Integrated nested Laplace approximations
LIC: Low-income countries
RR1: Relative risk of the first pre-crisis period (1999–2003)
RR2: Relative risk of the first pre-crisis period (2004–2008)
RR3: Relative risk of the first pre-crisis period (2009–2013)
