# Supplementary material for: Bayesian smoothed small-areas analysis of urban inequalities in fertility across 1999–2013
Source: Fertil Res Pract. 2019 Dec 21;5:17. doi: 10.1186/s40738-019-0066-8 (PMC6925428; doi:10.1186/s40738-019-0066-8)
Supplement: Supplementary file 1 — Additional file 1. Supplementary methods [file 40738_2019_66_MOESM1_ESM.docx]

**Supplementary methods.**

Statistical analysis

We analyzed fertility rate as the dependent variable. Fertility rate relies on population size. This because its variance is inversely proportional to the expected values. Then, those areas with low population tend to have larger estimated variability. To smooth the fertility rate, we used the hierarchical Bayesian model[1]. Those models assesses two types of random effects: spatial and heterogeneous. The former accounts for the spatial structure of the data. The latter deals with non-structural (non-spatial) variability. We used smoothed models to estimate the fertility rate for each period, mothers’ age group and nationality.

$$O_{i}\text{ \textasciitilde Poisson (}\mathrm{Pob}_{\mathrm{it}}\text{ }\theta_{\mathrm{it}}\text{)}$$

$$\mathrm{Log}\left( \theta_{\mathrm{it}} \right)= \alpha+S_{i}+H_{i}$$

We used maps to represent the geographical distribution of the smoothed fertility rate and the deprivation. All maps were generated using the R statistical package[2]. We analyzed the relationship between fertility rate and deprivation in the three periods. Then, we fitted an ecological regression model that accounts for the deprivation (D), the period (using two dummy variables, P_2_ and P_3_), and the interaction between deprivation and the dummy variables, as follows:

$$O_{i}\text{ \textasciitilde Poisson (}\mathrm{Pob}_{\mathrm{it}}\text{ }\theta_{\mathrm{it}}\text{)}$$

$\mathrm{Log}\left( \theta_{\mathrm{it}} \right)= \alpha+\beta_{1}D_{1}+\beta_{2}P_{2t}+\beta_{3}P_{3t}+\beta_{4}P_{2t}D_{i}+\beta_{5}P_{3t}D_{i}+S_{\mathrm{it}}+H_{\mathrm{it}}$

In that equation, for each area (i) and period (t) (t = 1 for the first pre-crisis period 1999-2003; t = 2 for the second pre-crisis period 2004-2008 and t = 3 for the crisis period 2009-2013):

O_it_ represents observed cases; Pob_it_ represents the female population in reproductive age; ϴ_it_ represents the relative risk with respect to the city; S_it_ represents the spatial effect, and H_it_ represents the heterogeneous effect. Lastly, P_2t_ and P_3t_ are dummy variables taking values: P_2t_ = 1 for t = 2, and t = 0 otherwise; and P_3t_ = 1 for t = 3, and t = 0 otherwise.

In both models, the spatial effect had an intrinsic conditional autoregressive prior distribution (ICAR) [1], which assumes that the expected value of each area coincides with the mean of the spatial effect of the adjacent areas and has variance of . The heterogeneous effect was represented by independent normal distributions with mean 0 and variance. The standard deviations and ^2^ were assigned a truncated-normal distribution with mean 0 and precision 0.0001. The parameters α and β_1_ to β_5_ were assigned a vague prior distribution.

As the deprivation index scale is non-dimensional and arbitrarily fixed for each period and city, we calculated the increase in risk that corresponds to a change in the deprivation index from its 5^th^ percentile value (P_5_) (low deprivation) to its 95^th^ percentile value (P_95_) (severe deprivation). This increase was calculated using the formula: $RR1=exp(\beta\left( P_{95}-P_{5} \right))$ for the first period, $RR2=exp(\left( \beta_{1}+\beta_{4} \right)\left( P_{95}-P_{5} \right))$ for the second period, and $RR3=\exp(\left( \beta_{1}+\beta_{5} \right)(P_{95}-P_{5}))$ for the third period. Finally, to address the ratio of RRs between the second period (pre-crisis) and the crisis period we used the equation: $RR23=exp(\left( \beta_{5}-\beta_{4} \right)\left( P_{95}-P_{5} \right))$. To analyze the previous trend in inequalities –before the crisis period- we computed: $RR12=exp(\left( \beta_{4} \right)\left( P_{95}-P_{5} \right))$. Relative risk (RR) estimates were based on the mean of their posterior distribution, and the corresponding 95% credible intervals (95% CI). These distributions were obtained using the “Integrated nested Laplace approximations” (INLA) method from the R statistical package (R.3.1.1)[3].

References

1. Besag J, York J, Mollie A. Bayesian image restoration, with two applications in spatial statistics. Ann Inst Stat Math. 1991;43:1–20.

2. The R Foundation. The R Project for Statistical Computing. The R Foundation; 2016. p. 1.

3. Rue H, Martino S, Chopin N. Approximate Bayesian inference for latent Gaussian models by using integrated nested Laplace approximations. J R Stat Soc Ser B (Statistical Methodol. Blackwell Publishing Ltd; 2009;71:319–92.
