# Supplementary material for: Bayesian smoothed small-areas analysis of urban inequalities in fertility across 1999–2013
Source: Fertil Res Pract. 2019 Dec 21;5:17. doi: 10.1186/s40738-019-0066-8 (PMC6925428; doi:10.1186/s40738-019-0066-8)
Supplement: Supplementary file 2 — Additional file 2. Supplementary contextual factors of the cities [file 40738_2019_66_MOESM2_ESM.doc]

**Supplementary contextual factors of the cities.**

| **Cities** | | **Total area^5^** | **Population^1^** | **Population^2^ (0-14)** | **Population^2^ (>65)** | **National population^2^** | **Immigrant population^2^** | **Birth rate^3^** | **Gross Mortality Rate^3^** | **Unemployment rate^2^** | **Average annual net income of households^1,4^** | **Average annual net income per habitant^1, 4^** |
| --- | --- | --- | --- | --- | --- | --- | --- | --- | --- | --- | --- | --- |
| **Biggest cities** | | | | | | | | | | | | |
|  | **Barcelona** | 102.76 | 1611822 | 12.36 | 21.14 | 82.55 | 17.45 | 8.72 | 9.54 | 18.03 | 36477.39 | 14946.29 |
|  | **Madrid** | 604.47 | 3207247 | 13.62 | 19.7 | 85.61 | 14.39 | 9.62 | 8.34 | 18.16 | 38539.41 | 15256.89 |
| **Northern** | | | | | | | | | | | | |
|  | **Bilbao** | 40.7 | 349356 | 12.21 | 22.73 | 91.88 | 8.12 | 7.78 | 10.08 | 19.52 | 39042.58 | 16291.05 |
|  | **San Sebastian** | 61.82 | 186500 | 12.77 | 21.73 | 92.93 | 7.07 | 7.66 | 9.91 | 12.55 | 39042.58 | 16291.05 |
|  | **Vitoria** | 277.04 | 241386 | 14.11 | 18.54 | 90.46 | 9.54 | 9.71 | 7.73 | 18.71 | 32848.02 | 16808.01 |
| **Southern** | | | | | | | | | | | | |
|  | **Almeria** | 296.07 | 192697 | 16.86 | 14.39 | 89.37 | 10.53 | 1.10 | 7.35 | 44.13 | 25867.69 | 9489.7 |
|  | **Cadiz** | 12.28 | 122990 | 12.48 | 20.4 | 97.99 | 2.01 | 6.91 | 10.65 | 35.45 | 29346.15 | 11131.85 |
|  | **Cordoba** | 1202.98 | 328704 | 15.88 | 16.84 | 97.13 | 2.87 | 9.54 | 7.93 | 40.1 | 27242.34 | 9991.65 |
|  | **Granada** | 87.98 | 238818 | 13.7 | 19.32 | 93.06 | 6.94 | 8.97 | 9.06 | 39.61 | 28305.19 | 11524.14 |
|  | **Huelva** | 156.06 | 148101 | 16.25 | 15.74 | 94.59 | 5.41 | 9.88 | 8.00 | 41.22 | 25672.46 | 9652.07 |
|  | **Jaen** | 424.01 | 116175 | 16.1 | 15.84 | 97.08 | 2.92 | 9.21 | 8.02 | 41.67 | 28837.01 | 10685.6 |
|  | **Málaga** | 395.4 | 568479 | 16.15 | 15.79 | 91.14 | 8.86 | 9.64 | 7.85 | 32.29 | 28922.43 | 9471.48 |
|  | **Sevilla** | 141.45 | 700169 | 15.14 | 17.58 | 94.39 | 5.61 | 9.52 | 8.59 | 32.08 | 28851.75 | 10829.41 |
| *Source: National institute of statistics of Spain. General year of reference: 2013.* | | | | | | | | | | | | |
| *^1^ Year of reference: 2015* | | | | | | | | | | | | |
| *^2^ Percentage* | | | | | | | | | | | | |
| *^3^ Per thousand people* | | | | | | | | | | | | |
| *^4^ In Euros* | | | | | | | | | | | | |
| *^5^ In Km^2^* | | | | | | | | | | | | |
