# Supplementary figures and images for: Bayesian smoothed small-areas analysis of urban inequalities in fertility across 1999–2013
Source: Fertil Res Pract. 2019 Dec 21;5:17. doi: 10.1186/s40738-019-0066-8 (PMC6925428; doi:10.1186/s40738-019-0066-8)

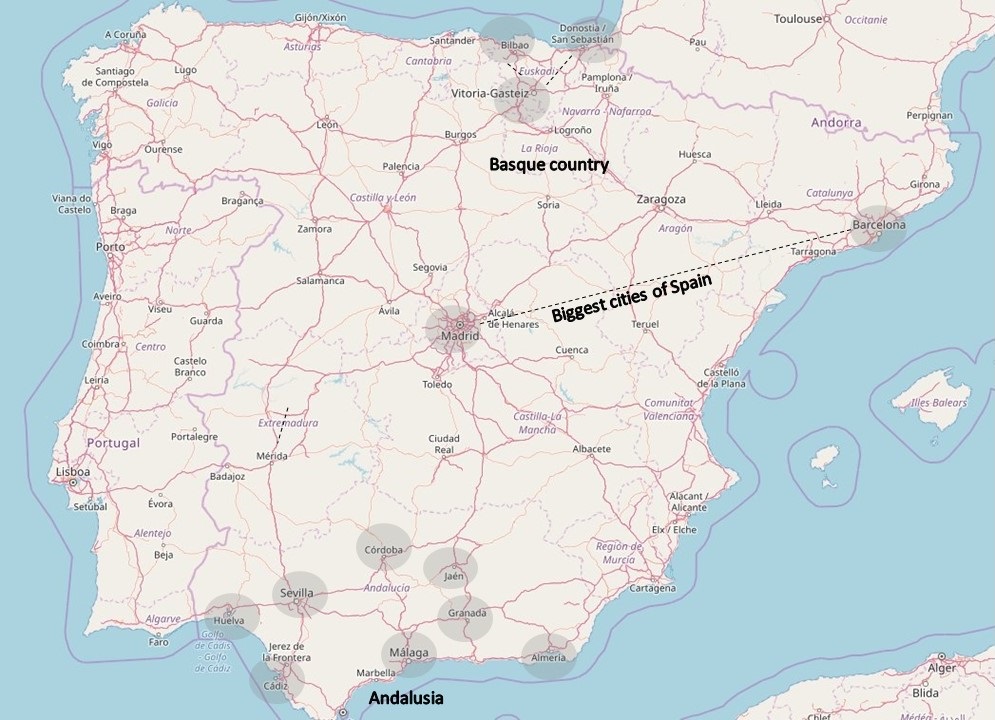

Supplement: Supplementary file 3 — Additional file 3. Supplementary file cities map [file 40738_2019_66_MOESM3_ESM.jpg]
